# Implications of the Actin Cytoskeleton on the Multi-Step Process of [*PSI*^+^] Prion Formation

**DOI:** 10.3390/v14071581

**Published:** 2022-07-21

**Authors:** Jane E. Dorweiler, Douglas R. Lyke, Nathan P. Lemoine, Samantha Guereca, Hannah E. Buchholz, Emily R. Legan, Claire M. Radtke, Anita L. Manogaran

**Affiliations:** 1 Department of Biological Sciences, Marquette University, Milwaukee, WI 53233, USA; jane.dorweiler@marquette.edu (J.E.D.); lykedr28@gmail.com (D.R.L.); nathan.lemoine@marquette.edu (N.P.L.); samantha.guereca@marquette.edu (S.G.); hannah.buchholz@marquette.edu (H.E.B.); emily.rose.legan@emory.edu (E.R.L.); claire.radtke@marquette.edu (C.M.R.); 2 Department of Zoology, Milwaukee Public Museum, Milwaukee, WI 53233, USA

**Keywords:** yeast prion, Sup35, actin cytoskeleton, Hsp104, protein aggregates

## Abstract

Yeast prions are self-perpetuating misfolded proteins that are infectious. In yeast, [*PSI*^+^] is the prion form of the Sup35 protein. While the study of [*PSI*^+^] has revealed important cellular mechanisms that contribute to prion propagation, the underlying cellular factors that influence prion formation are not well understood. Prion formation has been described as a multi-step process involving both the initial nucleation and growth of aggregates, followed by the subsequent transmission of prion particles to daughter cells. Prior evidence suggests that actin plays a role in this multi-step process, but actin’s precise role is unclear. Here, we investigate how actin influences the cell’s ability to manage newly formed visible aggregates and how actin influences the transmission of newly formed aggregates to future generations. At early steps, using 3D time-lapse microscopy, several actin mutants, and Markov modeling, we find that the movement of newly formed aggregates is random and actin independent. At later steps, our prion induction studies provide evidence that the transmission of newly formed prion particles to daughter cells is limited by the actin cytoskeletal network. We suspect that this limitation is because actin is used to possibly retain prion particles in the mother cell.

## 1. Introduction

Prions are self-perpetuating infectious aggregating proteins that are associated with fatal diseases in mammals and controllable heritable traits in yeast. The study of yeast prions has uncovered important cellular networks that contribute to the formation and maintenance of functional misfolded aggregates. Two of the most widely studied yeast prions are [*PSI^+^*] and [*PIN*^+^]. [*PSI*^+^] is the prion form of the translation termination factor Sup35 and [*PIN*^+^], also known as [*RNQ*^+^], is the prion form of the Rnq1 protein of unknown function. In both cases, the normally folded protein is converted to a misfolded conformation, which can subsequently convert additional normal protein molecules to the misfolded infectious form. Studies of [*PSI^+^*] and [*PIN^+^*] have uncovered that cytosolic chaperones play a pivotal role in prion propagation. Several chaperones, including the Hsp104 disaggregase, are critical for propagation by shearing preexisting large prion aggregates into smaller heritable particles [1,2,3,4]. While much is known about propagation, the mechanisms that underlie how prions are formed are less understood, including the role of cellular networks and chaperones in the initial formation of the prion.

Yeast prions spontaneously form at a very low frequency, generally observed in less than one in a million cells [5,6,7], making the study of spontaneous prion formation challenging. The process of “prion induction” has facilitated our understanding of formation. Overexpression of full length Sup35 or the N-terminal and middle domain of Sup35 (Sup35NM) can induce prions to form at a higher frequency [8,9,10]. Prion induction is further enhanced by the presence of [*PIN^+^*], which has been proposed to cross seed Sup35NM aggregation [11,12] or titrate Hsp104 activity in order to promote the formation of [*PSI*^+^] [13].

The process of [*PSI*^+^] induction can be monitored temporally by two different steps: the initial formation of aggregates in the mother cell that can be monitored by the presence of visible fluorescent aggregates and later transmission of prion particles to future generation that can be monitored through a colony growth assay. The initial visualization of newly formed aggregates is mediated through the fusion of a fluorescent marker to the Sup35 N and M regions (Sup35NM-GFP; [14]). Overexpression of Sup35NM-GFP leads to the appearance of small puncta that are transiently mobile for 20–30 min [15]. We have termed these small mobile puncta “early foci”. These early foci quickly grow and mature into static larger aggregates, which are located near the cell periphery [15,16,17]. The isolation of cells that contain these visible aggregates give rise to future generations that contain [*PSI*^+^] [15,18]. It is important to note that large visible aggregates are retained in the mother cell [16] and small non-visible aggregates or “propagons” are transmitted to daughter cells, thereby propagating the prion. 

It has been suggested that endocytic cortical actin patches, which are located near the cell membrane at sites of endocytosis, may play a role in prion nucleation. Gene deletions that alter endocytic cortical actin patch organization result in both reduced Sup35NM-GFP aggregate formation and lower prion induction frequencies [18,19,20,21,22]. However, the role actin plays in the two steps after nucleation, such as how the cell manages newly formed aggregates and how actin networks contribute to prion transmission, is poorly understood. To further dissect actin’s role in prion formation, we utilized several actin mutations and actin-inhibiting drugs to dissect how actin networks contribute to prion induction. Together, our results indicate that the mobility of early foci during the initial phases of formation is actin independent, but actin networks play a role in retaining aggregates as a single visible inclusion. Our prion induction studies also provide evidence that the transmission of newly formed prion propagons to daughter cells is linked to actin networks.

## 2. Materials and Methods

### 2.1. Strains, Plasmids, and Cultivation Procedures

Yeast strains in either the 74D-694 or BY4741 genetic backgrounds were used as indicated (Table S1). All strains were [*PIN*^+^] unless indicated in Table S1. Plasmids used in this study, with cross-reference to the figures in which they were used, are listed in Table S2. Yeast strains were grown using standard media and cultivation procedures [23]. Complex media containing 2% dextrose (YPD) or synthetic complete media containing the required amino acids and 2% dextrose (SD) was used as indicated. Strains transformed with plasmids were maintained on synthetic complete media lacking the specific amino acid. Several pharmacological drugs were used in this study. For temporary actin disruption in strains containing Sup35NM-GFP aggregates, cultures were treated with 100 μM latrunculin A (LatA), 100 μM of cytochalasin B (CytoB), or 25 μM of jasplakinolide (Jasp) for 30 min. Control strains were either untreated or treated with 50 μg/mL cycloheximide for 30 min. For longer treatment during [*PSI*^+^] induction studies, 1 or 2.5 μM LatA was added to overnight cultures grown in standard plasmid selective synthetic media. Higher concentrations lead to no cell growth. 

### 2.2. Sup35NM Early Foci and Aggregate Microscopy 

Strains were transformed with plasmids containing Sup35NM-GFP, Sup35NM-RFP, or Sup35NM-CFP, all of which are under a copper-inducible promoter (see Table S2). In 74D-694 strains, cells were grown in plasmid selective media supplemented with 25 μM copper sulfate for 16 h to efficiently time-lapse early foci, and for 24 h to score for the number of aggregates per cell. In BY4741 strains, cells were grown in plasmid selective liquid media supplemented with 50 μM copper sulfate for 24 h (approximate OD_600_ = 1–1.2) to time-lapse early foci and 48 h (saturated cultures) to score for the number of aggregates per cell. Images were captured with either a 63× (oil immersion, N.A. 1.4) or 100× (oil immersion, N.A. 1.44) objective on a DMI6000 Leica inverted microscope using Leica LAS X software (Leica Microsystems CMS GmbH, Germany, version 3.7.5.24914). For static 3D-images, 10–21 z-stack images were captured (the distances between individual z-stacks ranged from 0.2–0.8 μm). To ensure captured images were able to resolve the aggregate details for print, images were subjected to 3D deconvolution, using AutoQuant deconvolution algorithms (product of Media Cybernetics, Rockville, MD, USA) integrated within the LAS X software package, with background removed and intensity rescaled. All images shown are maximum projection unless indicated otherwise. 

Stains such as rhodamine-phalloidin, DAPI, and FM4-64 were added after induction in liquid media supplemented with copper, and immediately visualized by 3D microscopy to determine association of early foci or aggregate with stains. Similarly, co-localization of Sup35NM with fluorescently tagged proteins were visualized after induction in copper media. Pearson’s correlation coefficient was used to quantify co-localization between two fluorescently tagged proteins, as determined by the ImageJ Coloc2 plugin (ImageJ; collaborative product of National Institutes of Health and the Laboratory for Optical and Computational Instrumentation, University of Wisconsin, and is freely available in the public domain at https://imagej.nih.gov/ij/download.html, accessed on 1 July 2022). 

### 2.3. 3D Time-Lapse Microscopy and Coordinate Mapping 

Three-dimensional time-lapse microscopy of cultures was performed according to Sharma et al. [15]. Briefly, BY4741 cells were grown for 24 h in synthetic media supplemented with 50 μM copper sulfate. Then, 50 μL of culture was added to 300 μL of fresh selective media without copper and placed in concanavalin A coated Ibidi 1μ-slide 8 well glass bottom slides. Three-dimensional images were captured every 10 s over 45–300 min with 21–25 z-stacks (each step size was approximately 0.281–0.597 μm) using both brightfield and GFP filters and a 100× (oil immersion, N.A. 1.44) objective. To adjust for low Sup35NM fluorescence levels, videos were subjected to deconvolution to better resolve aggregate dynamics. Maximum intensity projection of z-stacks is shown.

Since particle tracking software had trouble tracking early foci because of the high diffuse cytoplasmic fluorescence, manual particle tracking was performed; “3D-coordinate mapping” involves plotting the x, y, and z coordinates of early foci in each image of a time lapse. This 3D-coordinate mapping was used to map the movement of early foci over time. The coordinates of the center of each early focus and also the coordinates of at least six edges of the cell membrane were recorded. Coordinates were verified by two separate investigators. Raw x, y, and z coordinates for each focus over 100 frames were entered into Python to generate three-dimensional traces (Figure S4C). These measurements were used to calculate distances moved per frame and speeds between two time points. “Rolling” or simple moving average (SMA) speed per 30 s was determined by averaging the speed of three successive 10 s recordings over time in order to smooth data for analysis. Simple linear regression of SMA was used to understand overall speed trends over time. 

To further understand early foci behavior, we borrowed from dynamic models established in studying movement in animal ecology. These models were used to draw inference into how early foci in wildtype cells move within the cellular environment to determine (1) whether early foci movement is random and (2) understand the variance in movement over time. Specifically, we applied a Markov movement model to understand early foci movement [24,25]:

y_t+1_ = y_t_ + γ_s_T(θ_s_)d_t−1_ + Σ_s_

where y_t_ and y_t+1_ are the location of early foci in two subsequent steps, γ_s_ is the degree of autocorrelation with the previous movement vector (d_t−1_), T is a rotation matrix for the average turn angle (θ_s_), and Σ_s_ is the random walk displacement matrix. Random movement is determined by γ_s_ values, where values close to zero indicate movement is random and values close to one indicate near perfect correlation with the previous movement. Random walk displacement is determined by (Σ), with values close to zero indicating that movement is not variable (i.e., traveling the same distance at each step) and higher values indicating large variability in movement between time points (i.e., traveling very different distances between time points). To determine the volume of early foci and the cell, measurements were taken using LAS X software to measure the length of the focus or cell in the x, y, and z directions. The three distances were used to determine the volume of an ellipsoid (V = 4/3 pabc) for each early focus over 100 frames. All Python codes and files have been deposited in figshare: (https://doi.org/10.6084/m9.figshare.19661001.v1; accessed on 26 April 2022). We used linear regression to determine if early foci volume or early foci:cell volume was linearly related to either the correlation of movement (γ) or variability in movement (Σ).

To determine the position of aggregates within the cell, 3D images with 21-step z-stacks imaged in both brightfield and the fluorescent channel were acquired. The x, y, and z coordinates, as well as the diameter of the cell in four separate directions, were recorded. The diameters were used to determine the average cell radius to draw a conceptual sphere. This conceptual sphere represents the mathematical periphery of the cell. The relative coordinates of the aggregate were plotted within the conceptual sphere and the relative distance of the aggregate from the cell periphery was determined (where 0.0 is considered the cell periphery and 0.5 is considered the middle of the cell). 

### 2.4. Western Blot Analysis

Cell lysates were prepared from 50 mL of overnight cultures and lysed according to Sharma et al. (2017). Then, 100 μg of crude lysates were treated with 2% SDS-Buffer and boiled before loading SDS-PAGE gels. All gels were transferred onto PVDF and immunoblotted with anti-GFP antibody (to detect Sup35NM-GFP), anti-Sup35C (to detect endogenous Sup35), and anti-PGK. 

### 2.5. De Novo [PSI^+^] Induction

To perform [*PSI*^+^] induction experiments, [*PIN*^+^] BY4741 strains were transformed with *pCUP-SUP35NM-GFP* plasmid (*HIS3*, p3031) and the *ura3-14* [*PSI*^+^] suppressible allele (LEU2; p3107; [26]). The *ura3-14* allele allows scoring of colonies that are [*PSI*^+^] after induction, since only [*PSI*^+^] cells grow on SD-Leu-Ura media. For each wildtype and mutant BY4741 strain, at least three fresh transformants were grown in selective synthetic media (SD-His-Leu) supplemented with 50 µM CuSO_4_ for 40–46 h. Strains were then observed under the fluorescent microscope to verify the presence of newly formed fluorescent aggregates. Approximately 100 cells were plated on SD-Leu and 100-fold higher cell density was plated on SD-Leu-Ura. [*PSI*^+^] induction frequency was calculated based upon the number of colonies formed on SD-Leu-Ura plates divided by the total number of CFUs plated, as determined by the number of colonies on SD-Leu. There was negligible growth on SD-Leu-Ura from cultures that were not exposed to copper sulfate. For actin drug analysis, 74D-694 strains were used and plated on rich media (YPD) after drug treatment. [*PSI^+^*] colonies range from dark pink to white, whereas [*psi*^−^] colonies are red. Since rich media provides the ability to score for a wider range of prion variants than the *ura3-14* allele, induction frequencies are usually higher. 

## 3. Results

### 3.1. Co-Localization between Sup35NM-GFP Early Foci and Actin Networks Is Inconclusive

Our previous work showed that transient overexpression of Sup35NM-GFP leads to the formation of small puncta that are mobile for approximately 20–30 min, which we termed “early foci” [15]. Using time-lapse microscopy, we observed temporal progression of smaller, mobile early foci to larger, less mobile “aggregates” that remain localized near the cell periphery. Consistent with previous observations [18,21], occasional co-localization was observed between the actin label, rhodamine-phalloidin, and static Sup35NM-GFP aggregates (Figure S1). However, the fixation process appeared to alter the aggregate morphology. To determine whether actin co-localizes with both early foci and aggregates, while avoiding fixation, we turned to 3D-live cell imaging using fluorescently tagged yeast actin-binding proteins. Filamentous actin networks consist of both actin patches and actin cables. A Cof1-RFP fusion protein, which localizes to cortical actin patches [27], was used to screen for co-localization. Sup35NM-GFP was overexpressed for 16 h to capture early foci, or for 24 h to image aggregates. Capturing early foci is difficult because the temporal progression to aggregates rarely exceeds 20–30 min [15]. Therefore, a tremendous amount of screening is required in order to image early foci. We did not observe any clear examples of co-localization between Sup35NM-GFP and Cof1-RFP (Figure S2A). In contrast to early foci, Sup35NM-GFP aggregates are easy to capture. Despite analyzing many cells, we rarely observed co-localization between Cof1-RFP-labeled actin patches and Sup35NM-GFP aggregates (Figure S2A,C). Actin filaments can also assemble into long bundles called actin cables, which are necessary for cell polarity and polarized particle movement. An ABP140-YFP fusion protein, which is associated with actin cables [28,29], showed very little co-localization with early foci or aggregates (Figure S2B,C).

### 3.2. Actin Polarization Mutants form Multiple Sup35NM-GFP Aggregates 

Our previous studies were performed in the 74D-694 genetic background because this strain contains a [*PSI*^+^] suppressible marker to assess the prion state of the cell [15,19,30]. We noticed that the 74D-694 strain developed multiple aggregates per cell compared to the BY4741 strain (Figure S3), a common laboratory wildtype genetic background. Based on these observations, we performed all subsequent studies in the BY4741 strain because the formation of a single focus or aggregate was more tractable to study than multiple. 

Even though we did not see co-localization between actin-binding proteins and fluorescently tagged Sup35NM early foci or aggregates, we and others have reported a connection between prion aggregate formation and an array of actin-associated proteins [18,19,21]. Therefore, we used a genetic approach to understand the link between prion formation and actin networks, by using several actin point mutants. We used four previously characterized actin mutants [31,32] that show actin patch polarization defects (*act1-120*, *act1-122)*, actin cable defects (*act1-101*), and a double point mutant that alters nucleotide binding (*act1-129*: R177A and D179A). It should be noted that another study showed that the single R177A mutation limits aggregate formation [18]. 

**Figure 1:**
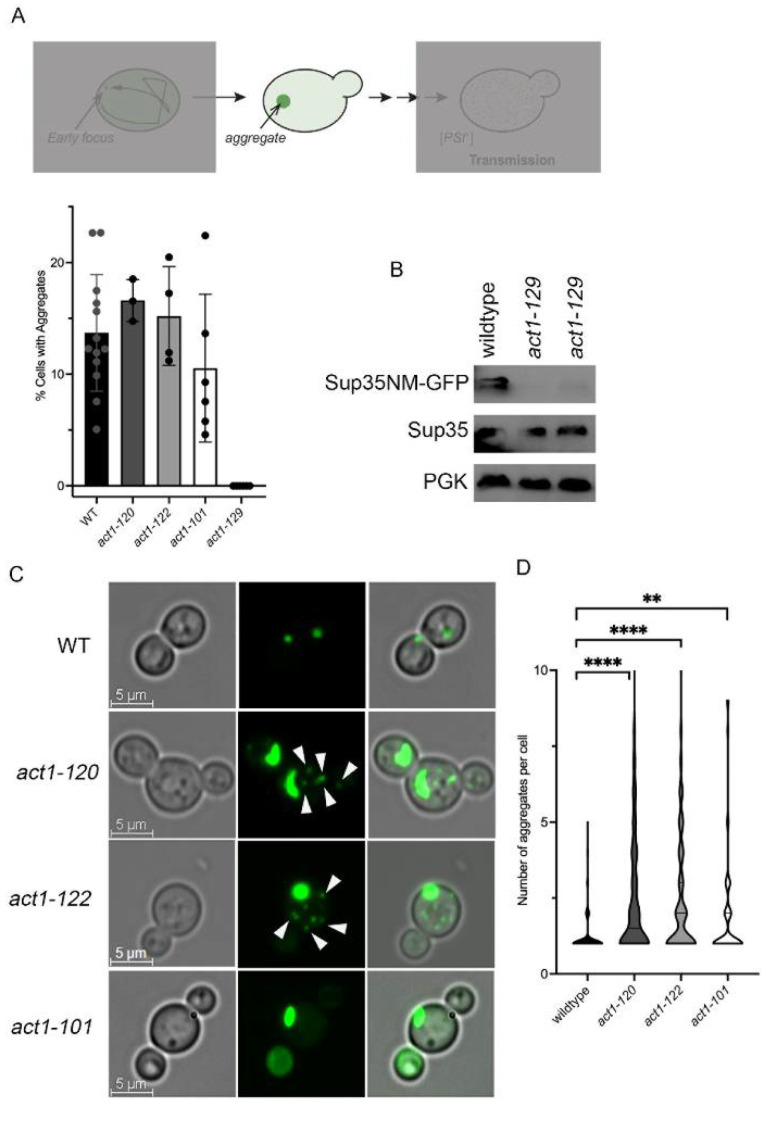
Multiple Sup35NM-GFP aggregates are detected in *act1-120* and *act1-122* strains. (**A**) Top, model showing multiple steps of [*PSI*^+^] formation analyzed in this paper and highlighting the aggregate step as the primary focus of this figure. Bottom, BY4741 strains containing Sup35NM-GFP were grown in the presence of copper for 48 h to assess the formation of aggregates. Each dot represents at least 50 cells from an individual transformant (six transformants were screened for *act1-129*). Data are shown as means ± SD. Note that *act1-129* strains showed low visible GFP signal. (**B**) Western blot analysis of two independent *act1-129* transformants expressing Sup35NM-GFP for 24 h. Anti-GFP (top), anti-Sup35C (to detect endogenous Sup35), and anti-PGK antibodies were used. (**C**) Bright field, GFP, and merged images of the indicated strains are shown. Arrows indicate the small visible aggregates formed in *act1-122* and *act1-120* strains. (**D**) The number of aggregates per cell was assessed in the indicated strains. Data shown as a violin plot to indicate the number of aggregates per cell observed. Each plot represents a total of 66–248 cells from at least three independent experiments. ** *p* < 0.001 and **** *p* < 0.0001 values by Welch’s *t*-test.

Wildtype, *act1-120*, *act1-122*, and *act1-101* strains all had similar percentages of cells that contained aggregates, indicating that these mutations do not impact the formation of the aggregates. However, *act1-129* never formed any aggregates (Figure 1A) and had low visible GFP levels. Using Western blot analysis, the Sup35NM-GFP steady-state levels in *act1-129* strains were very low, whereas endogenous Sup35 protein levels (anti-Sup35C) appeared normal (Figure 1B). The lack of Sup35NM-GFP expression likely explains the lack of aggregates and, accordingly, *act1-129* was not pursued in subsequent studies.

While there were no differences in the percent of cells that contain aggregates, we wanted to know whether the aggregates were different between actin mutants and wildtype strains. We found a significant distinction in the number of aggregates per cell between the two actin mutants with actin patch polarization defects (*act1-120* and *act1-122*) relative to WT strains. Morphologically, 88% of wildtype cells contained a single aggregate (Figure 1C,D), whereas less than 50% of *act1-120* and *act1-122* cells contained a single aggregate. The remaining proportion of cells in these actin mutants contained multiple aggregates, typically observed as a distinct large aggregate and many smaller aggregates (Figure 1C, arrows). Analysis of the number of aggregates found in cells indicates that *act1-120* and *act1-122* had significantly more aggregates per cell than wildtype (Figure 1C,D). *act1-101* appeared to be intermediate, with approximately two-thirds of the cells containing a single aggregate. 

### 3.3. Early Foci Size Is Correlated with Movement Variability, but Early Foci Movement Is Random

**Figure 2:**
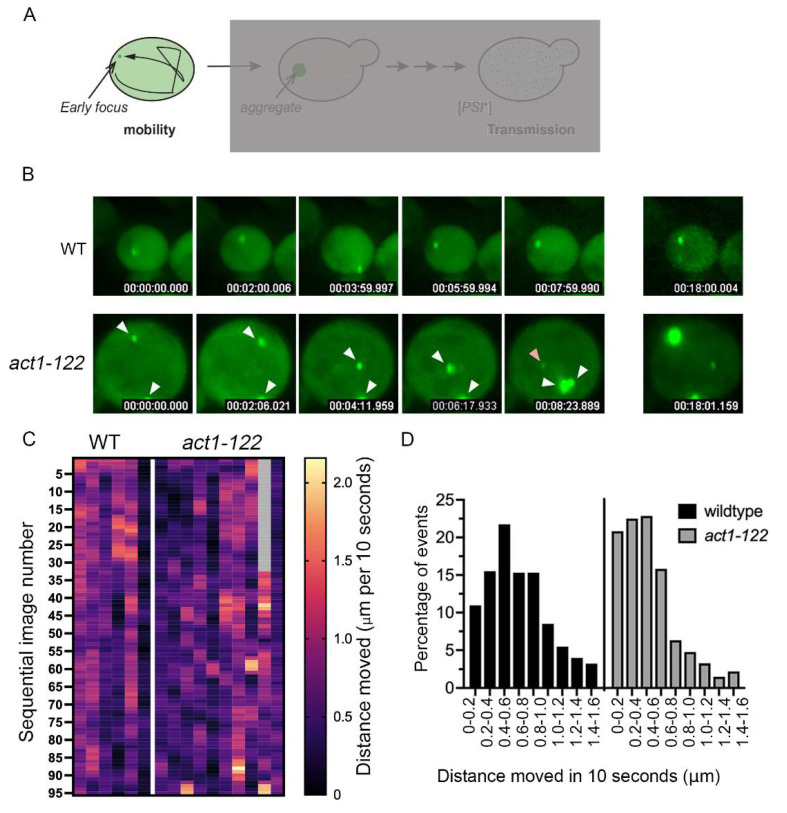
Multiple early foci form in *act1-122* mutants and appear to be generally less mobile. (**A**) Top, model showing the multiple steps of [*PSI*^+^] formation analyzed in this paper and highlighting the early foci step analyzed in this figure. (**B**) Sup35NM-GFP was expressed for 16–24 h with copper, with the indicated strains transferred into deep well slides containing fresh media without copper. Cells with early foci were imaged every 10 s in three dimensions. White arrows indicate multiple foci and pink arrow indicates a newly appearing focus. Images are maximum projections of 3D images. (**C**) By plotting 3D coordinates of foci within each image, the distance moved (μm per 10 s) is plotted on the heat map. Each column represents the distance moved of an individual focus (even if there are multiple aggregates per cell, as indicated by arrows in (**B**)). The second column from the last in the *act1-122* heatmap corresponds to a new focus indicated by the pink arrow in (**B**) above, such that the first 30+ timepoints are indicated as no data. (**D**) The distance traveled (per 10 s) distribution for early foci movement is shown in wildtype (**left**) or *act1-122* (**right**) strains. The run length, or cumulative distance traveled in 10 s, was compiled for all early foci in the indicated strains. Histograms show the distribution of run lengths vs. the percentage of times that length was observed.

The increase in the number of aggregates formed in *act1-122* and *act1-120* cells suggests that there may be something different in the formation process. Therefore, we performed 3D time-lapse microscopy to study the formation of early foci. Because various actin mutants are temperature sensitive, we sought to avoid any possible confounding effects of growth rate. Thus, *act1-122* strains were chosen because the strain grows well compared to other actin mutants at permissive temperatures [31]. Sup35NM-GFP was induced and imaged at room temperature to capture early foci maturation. As expected, early foci in wildtype cells were mobile for approximately 20–30 min (Figure 2 and Video S1). Early foci in *act1-122* cells were slightly different. While early foci were similarly mobile in *act1-122* cells, the number of foci per cell rarely decreased (Figure 2B and Video S2). These results likely explain the multiple aggregates observed per *act1-122* cell at later time points (Figure 1D).

In order to quantitatively study early foci dynamics in *act1-122* over time, we plotted the three-dimensional position of each early focus found in either wildtype or *act1-122* cells. Specifically, x, y, and z coordinates of each focus were recorded every 10 s over 16 min (100 frames) for five individual early foci from five separate wildtype cells (Video S1) and 11 early foci from six individual *act1-122* cells (Video S2). Heatmap analysis of individual early foci with both wildtype and *act1*-*122* cells suggest that wildtype early foci generally move more rapidly than foci in *act1-122* cells (Figure 2C). On average, the speed of early foci was approximately 75 nm/s in wildtype cells and 55 nm/s in *act1-122* mutants within the first 16 min of initial appearance. To further understand the distribution of distances traveled by each early focus, we tallied the net distances traveled (or run length) in 10 s for both strains. While the majority of run lengths of early foci in wildtype cells ranged from 200–1000 nm in 10 s, early foci in *act1-122* cells ranged from 0–600 nm in 10 s (Figure 2D). Taken together, wildtype early foci tend to travel longer distances per minute than *act1-122* early foci. 

**Figure 3:**
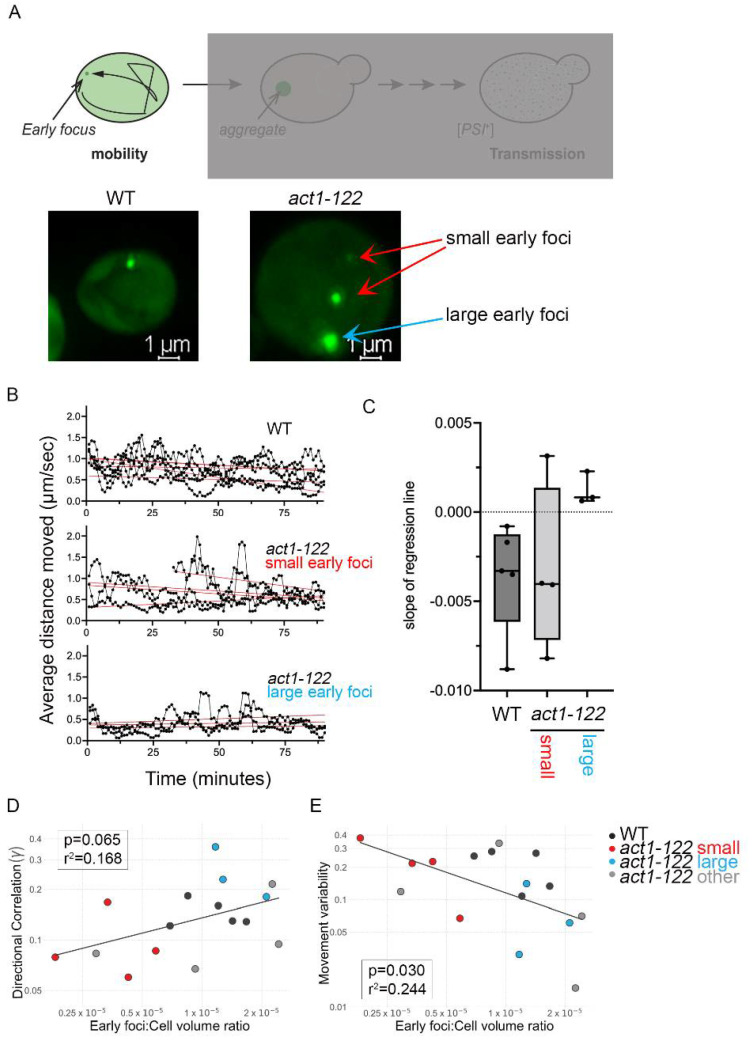
Small early foci in *act1-122* strains are more mobile than large foci. (**A**) A representative image of a wildtype cell with one early focus versus a representative image of an *act1-122* cell with multiple foci. Note that one early focus is larger than the rest in the *act1-122* cell. (**B**) Using the 3D coordinates and distance moved per 10 s time interval presented in Figure 2, the simple moving average (SMA) speed of each early focus per 30 s (the average of speed taken between three subsequent frames) was plotted over time in wildtype cells (**top**). The SMA speed of small early foci (**middle**) or large early foci (**bottom**) from *act1-122* strains were plotted over time. Red lines indicate the simple linear regression line of each individual early focus. A total of 11 early foci were tracked among the 6 *act1-122* cells, but 4 such foci could not be cleanly categorized as small vs. large (Figure S4), so are not included within any of the plots of (**B**). However, they are included below in parts (**D**,**E**) of this figure as “other.” (**C**) The slope of the red regression lines in (**B**) for each early focus (wildtype, *act1-122* small and large early foci) are displayed as box and whisker plots with median and interquartile ranges. Dots represent the slopes of foci regression lines indicated in (**B**). (**D**) Directional correlation measures randomness, with values above 0.5 indicating non-random movement. Directional correlation is plotted against early foci:cell volume ratio. (**E**) Movement variability is plotted against early foci:cell volume ratio. The colors indicate data points from specific foci categories. Dots in (**D**,**E**) correspond to each of the 16 early foci tracked in wildtype and *act1-122* cells, and are color coded according to the legend at right (**E**), with "other” representing those foci in *act1-122* cells that could not be cleanly categorized as small vs. large.

Time-lapse analysis of *act1-122* cells also showed that when multiple early foci are formed, one was usually very large and the others were smaller (Figure 3A and Figure S4). Interestingly, it appeared that the smaller foci were more mobile. To understand mobility, we plotted the simple moving average speed over time for wildtype early foci and *act1-122* small and large early foci (Figure 3B). Simple linear regression lines were added to visualize the movement trend for each focus, revealing both the initial degree of movement (y-intercept of regression lines) and the degree to which that movement changed over time (slope of regression lines). The higher y-intercepts for both wildtype and small *act1-122* foci demonstrate higher initial mobility relative to the large *act1-122* foci (lower y-intercepts; Figure 3B). When comparing slopes between the three different categories, wildtype and small *act1-122* foci slowed down over time as demonstrated by the negative slopes. Conversely, the slopes of large *act1-122* foci regression lines are close to zero, suggesting no significant change in their mobility once they appear (Figure 3C). Taken together, *act1-122* strains form multiple early foci, in which smaller foci have more movement than larger foci.

Since actin plays an important role in cargo movement in yeast, we speculated that early foci movement may use actin networks to move directionally in the cell. To draw inference in whether early foci movement in wildtype and *act1-122* is directed or random, we applied a Markov movement model by using the positional values of the early foci over time and determining whether (a) the movement of early foci is random (directional correlation) and (b) whether foci exhibit different types of movement using random walk displacement (movement variability). All early foci, regardless of whether from wildtype or *act1-122* cells, showed a directional correlation below 0.4 (Figure 3D and Figure S4C), indicating that movement is random. When directional correlation was plotted against focus size (specifically volume), we observed a positive correlation (*p* = 0.010; r^2^ = 0.385) indicating that larger foci display less randomness than small foci (Figure S4A–C). Conversely, when plotting foci size against movement variability, a negative correlation (*p* = 0.088; r^2^ = 0.193) was observed (Figure S4D) indicating that smaller foci have erratic movement patterns while larger aggregates do not move much. While the latter was not significant, these data suggest there is a correlation between foci size and movement. 

If movement is random, and variability of movement is based on size, we speculated early foci in large cells may have more movement variability than when in the confines of a smaller cell. Therefore, we plotted the ratio of early foci volume relative to the volume of the cell and compared these values with directional correlation (randomness) and movement variation. When plotting randomness to early foci:cell volume ratio, there was a positive correlation (*p* = 0.065; r^2^ = 0.168; Figure 3D), while movement variation to early foci:cell volume ratio showed a significant negative correlation (*p* = 0.030; r^2^ = 0.244; Figure 3E). As we proposed, these data suggest that movement of small early foci is much more variable and less directed when cells are larger. However, it is important to note that larger foci compared to cell volume show higher directional correlation and lower movement variability (Figure 3D,E) regardless of the genetic background. 

### 3.4. Sup35NM Aggregates Do Not Localize with Organelles or IPOD

We previously reported that newly formed early foci are initially mobile but then slow after 20–30 min. The temporal progression from early foci to aggregates show that aggregate movement is static over several hours of time-lapse [15]. To understand where these aggregates reside shortly after initially forming, we performed staining and co-localization experiments. Newly formed fluorescently-tagged Sup35NM aggregates did not co-localize with nuclear or vacuole stains, and did not co-localize with ER, mitochondrial, or peroxisomal-associated proteins (Figure S5A–C). While previous work suggested that Sup35NM aggregates are localized to IPOD [33,34], our experiments failed to detect any consistent localization of Sup35NM-GFP near vacuoles. To further explore localization, we assessed whether fluorescently tagged Sup35NM co-localized with previously characterized IPOD marker Rnq1-GFP [35] and the nearby pre-autophagosome structure, Atg8 [34,35,36]. We did not observe co-localization with either protein (Figure S5D), suggesting that aggregates are not localized to IPOD or pre-autophagic structures, even several hours after initial formation.

### 3.5. Hsp104 Co-Localizes to Early Foci Intermittently

**Figure 4:**
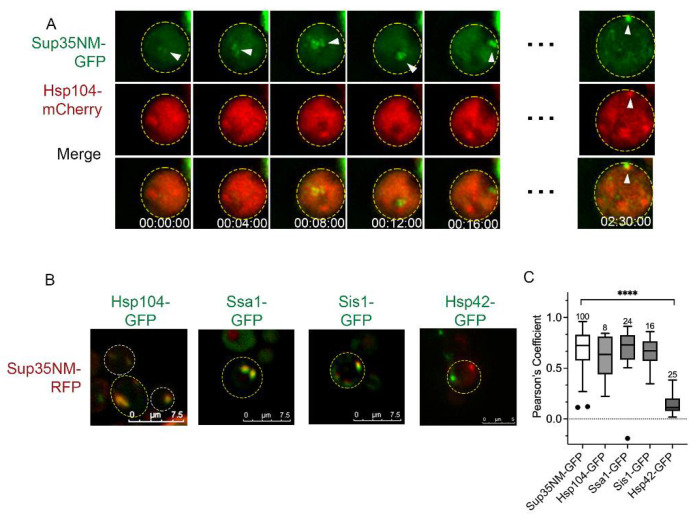
Sup35NM-GFP aggregates are associated with Hsp104, Sis1, and Ssa1. (**A**) 3D Time-lapse microscopy of early foci in wildtype cells expressing Hsp104-mCherry. (**B**) Co-localization of Sup35NM-RFP aggregates (shown in green) and indicated GFP proteins (red). (**C**) Pearson’s correlation coefficient was used to quantify the degree of co-localization between Sup35NM-RFP and the indicated proteins. Note that co-localization between Sup35NM-GFP and Sup35NM-RFP is used as a control. Data shown as box and whisker plots with medians and interquartile ranges, and number of cells analyzed above each box. Dots indicate outliers as identified by Tukey analysis. Only Hsp42-GFP is significantly different from controls as determined by Welch’s *t*-test. **** *p* < 0.001.

Hsp104 has been shown to co-localize with Sup35NM-GFP aggregates [17], yet it is unclear whether Hsp104 is associated during the formation of early foci. Here, we expressed Hsp104-mCherry in Sup35NM-GFP-induced strains and performed 3D-timelapse microscopy. Co-localization was inconsistent between Hsp104 and Sup35NM early foci. Upon initial formation, some videos showed no co-localization (Figure 4A) while other videos showed sporadic localization, as if the Hsp104 signal was chasing the Sup35NM signal over time (Figure S6A). However, once aggregates were well established, co-localization between Hsp104 and Sup35NM-GFP aggregates was very consistent (Figure 4B,C). These results imply that early foci form first and Hsp104 is subsequently recruited to these aggregates.

We also looked at co-localization between Sup35NM-GFP and other chaperones. Ssa1 and Sis1, both Hsp104 co-chaperones, consistently co-localize with Sup35NM-GFP aggregates (Figure 4B,C). Hsp42, which has been shown to associate with heat-induced aggregates and possibly play a role in their protein sequestration [37,38,39], showed no co-localization with early foci (Figure S6B) or established aggregates (Figure 4B,C). While heat-induced peripheral aggregates fail to form in *hsp42D* cells [37], Sup35NM-GFP aggregates form normally in these strains (Figure S6C). Taken together, our data suggests that early foci possibly form in an Hsp42 independent fashion and Hsp104 is recruited shortly thereafter. Based on the work of others who have shown localization between Sup35NM-GFP aggregates and IPOD [16,34], we suspect that Sup35NM aggregates may be trafficked to IPOD much later as the cell ages. 

### 3.6. Actin Fibril Disruption Changes Aggregate Position

**Figure 5:**
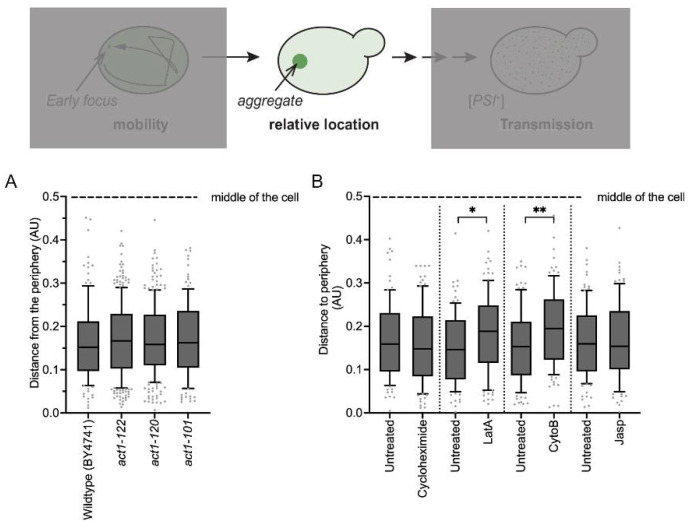
Disruption of actin networks alters Sup35NM-GFP aggregate position relative to the cell membrane. (**A**) The position of Sup35NM-GFP aggregates was determined by 3D-coordinate mapping to determine the relative distance of the aggregate to the cell membrane (where 0 is the cell membrane and 0.5 is the middle of the cell). Aggregate distance from the periphery was scored in the indicated BY4741 wildtype or actin mutant strains. Data shown as box and whisker plots with medians, interquartile ranges, and dots indicate outliers by Tukey analysis. Each boxplot represents a total of at least 125 cells from a minimum of four trials. No values were significant by Welch’s *t*-test. (**B**) 74D-694 wildtype Sup35NM-GFP cultures were grown for 24 h in the presence of copper. Parallel cultures were either treated with ethanol (untreated) or cycloheximide, latrunculin-A (LatA), cytochalasin (CytoB), jasplakinolide (Jasp) for 30 minutes prior to imaging. Data are shown as box and whisker plots with medians, interquartile ranges, and dots indicate outliers by Tukey analysis. Each boxplot represents a total of at least 95 cells from three trials. Individual treatments were compared to the corresponding paired control by a paired *t*-test (* *p* < 0.01, ** *p* < 0.001). Note that the distribution of values increased significantly upon LatA and CytoB treatment as determined by Kolmogorov–Smirnov test (*p* < 0.01).

We next asked whether the disruption of actin changes the position of the mature aggregate within the cell. While our co-localization studies did not provide a reliable marker to monitor cellular localization of aggregates, we relied on the relative location of the aggregate from the cell membrane since previous reports suggested that Sup35NM-GFP aggregates are initially localized near the cell periphery [15,33]. We established a quantitative method to map the location of the aggregate within the cell relative to the cell membrane. The 3D x, y, and z coordinates of the aggregate position were mapped, as well as positional location of several points on the cell membrane. Based on radii measurements of the cell, a conceptual sphere was generated, with geometrical derivations used to estimate the relative distance the aggregate was from the cell periphery. We found that aggregates in wildtype cells were biased near the cell membrane (Figure 5A). Furthermore, the relative position of aggregates in three separate actin mutants were no different from the positions of wildtype aggregates (Figure 5A). However, if aggregate location within the cell is dependent upon properly formed actin networks, then more blatant disruption of actin networks would be required. Therefore, we used three actin-inhibiting drugs. Latrunculin A (LatA) disrupts microfilament organization, cytochalasin B (CytoB) inhibits actin polymerization, and jasplakinolide (Jasp) polymerizes and stabilizes actin filaments. When cells were briefly treated with the protein synthesis inhibitor, cycloheximide, there was no change in aggregate position. Brief treatment of actin drugs LatA or CytoB resulted in a significant change in aggregate position compared to paired untreated controls. Specifically, these treatments, which targeted microfilament organization or polymerization, resulted in an increased distribution of aggregate position between the cell membrane and the middle of the cell. Jasp, which fosters actin filament polymerization and stabilization, had no effect. Together, these data indicate that disruption of actin polymerization or filaments is associated with altered aggregate position.

### 3.7. act1-122 and act1-101 Significantly Alter Prion Induction Frequency

**Figure 6:**
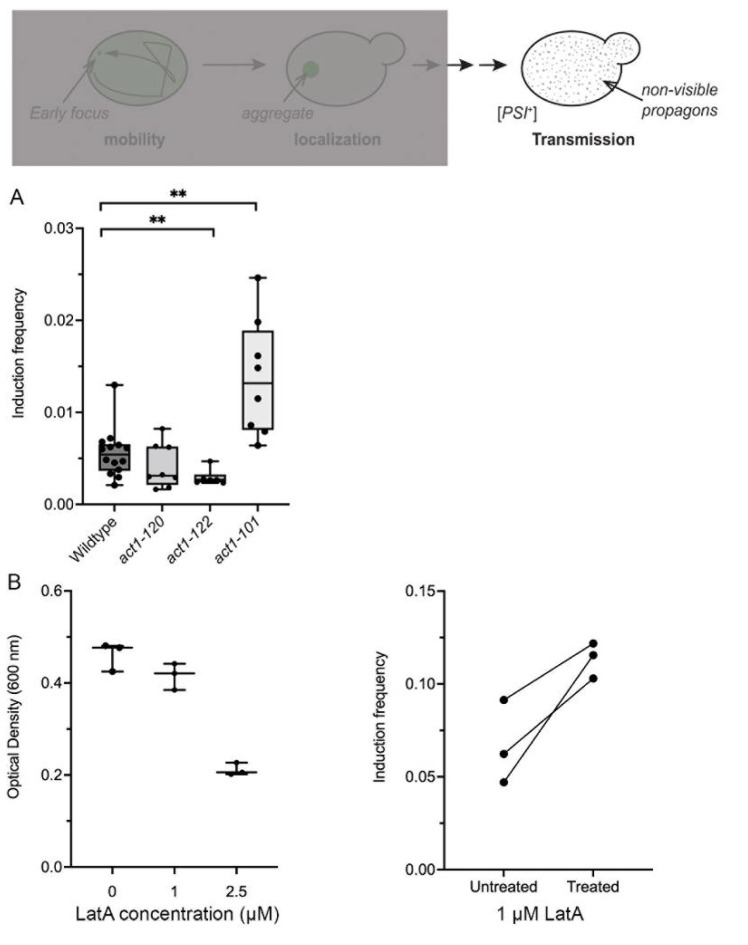
*act1-122* strains have reduced prion induction frequency, while *act1-101* and LatA-treated strains have increased induction frequency. The diagram shows that non-visible propagons (gray dots) from the original aggregate are transmitted to daughter cells to propagate [*PSI*^+^]. (**A**) Sup35NM-GFP was induced for 48 h in the indicated backgrounds, with cells plated on SD-Leu-Ura to score for the ability to transmit prion particles to subsequent generations. This passage is scored by prion induction frequency. Dots indicate raw values of individual trials. ** *p* < 0.001 by Welch’s *t*-test. (**B**) Left panel: 74D-694 wildtype strains were treated with the indicated concentrations of LatA and optical densities were assessed after 24 h of growth to determine whether overnight growth is inhibited. Right panel: Sup35NM-GFP was induced in paired untreated and treated cultures with 1 μM LatA for 24 h, and then plated on rich media to determine induction frequency (see Section 2). Although each treated culture exhibits higher induction frequency relative to its untreated pair, the overall effect remains insignificant (*p* = 0.055 by paired *t*-test). Dots indicate raw values of individual trials.

Based on the data above, it appears that the genetic manipulation of actin networks influences the number of aggregates formed but does not directly impact the mobility of these aggregates. However, it was unclear how actin networks influence the transmission of non-visible propagons to daughter cells for propagation of the prion (Figure 6). To address this question, we introduced a *ura3-14* [*PSI*^+^] suppressible marker [26] into actin mutants to score for the [*PSI*^+^] induction. Sup35NM-GFP was overexpressed in wildtype and the three *ACT1* mutants, with the formation of visible aggregates confirmed by microscopy, similar to Figure 1A. Once confirmed, strains were plated and scored for [*PSI*^+^] induction frequency. Wildtype strains had an average induction frequency of 0.006, which is similar to our previous induction frequency in the 74D-694 genotype [19]. First, we looked at *act1-120* and *act1-122,* which both contain mutations in the same region of actin and display depolarized actin patches [31]. Induction frequencies in *act1-120* strains were similar to wildtype strains, yet frequencies in *act1-122* were 1.7-fold less than wildtype. Next, we looked at *act1-101*, which have disrupted actin cables [32,40] yet grow normally at permissive temperatures [31]. *act1-101* strains exhibited an average induction frequency of 0.014, which was almost 2.5-fold higher than wildtype cells. However, we did observe frequencies as high as 0.25 in *act1-101* mutants. 

These results suggest that the disruption of cable networks increase the ability of propagons to be transmitted to daughter cells for prion propagation. We also tested induction frequency in the presence of latrunculin A, which disrupts actin cables. We found that very low concentrations of LatA did not disrupt cell growth (Figure 6B), but appeared to modestly, but not significantly (*p* = 0.055), increase prion induction frequency compared to paired untreated control cultures. However, it is important to note that the treatment of LatA in all three independent paired cultures lead to an increase in prion induction frequency, suggesting that actin cable disruption increases the conditions in which newly formed prion aggregates can be propagated.

## 4. Discussion

Here, we used fluorescently tagged Sup35NM-induced prion formation to understand how cells manage newly formed aggregates and how actin networks influence this process. Using several actin mutants and pharmacological disruptors, our study shows (a) early foci movement is random (Figure 3), (b) some actin mutants form a higher number of aggregates per cell (Figure 1), (c) aggregates appear to localize near the cell periphery but localization changes upon treatment with actin depolymerizing drugs (Figure 5), and (d) prion transmission is altered in some actin mutants (Figure 6).

### 4.1. Mobility of Early Foci and Localization

We traced early foci formation to understand how cells manage newly formed inclusions. Using 3D coordinate mapping and Markov models, we showed that early foci movement is random, regardless of genetic background (Figure 3), thus indicating that movement is not directed, and thereby actin independent. Interestingly, other cytosolic entities have been shown to have similar behavior. Q-bodies are characterized as cytosolic compartments that form in response to the temporary storage of misfolded proteins prior to degradation. Newly formed heat-induced Q-bodies are also mobile and move in a random fashion [38]. While our observations showed that early foci moved a little faster than Q-bodies (50–80 nm/s vs. 5–15 nm/s), the movement of both inclusions are random. However, it is important to note that while Q-bodies are Hsp42 dependent, Sup35NM-GFP early foci formation is not (Figure 4B,C and Figure S6). 

Based on our modeling studies, we observed a correlation between the variability in movement and early foci size. Where small early foci showed erratic variable mobility, large early foci had very little movement. One possible explanation for these observations is that within the crowded environment of the cytoplasm, space is restricted. Small foci will naturally go to open space and respond to the cytoplasmic circulation of fluid, which likely corresponds to the observed variable movement. Larger aggregates, due to the increased size and limited space, are less likely influenced by cytoplasmic streaming. This possibility is supported by our data that shows movement variability is higher when the early foci:cell volume ratio is low (i.e., small foci are housed in a large cell; Figure 3E). 

Early foci in both wildtype and *act1-122* strains were mobile for the first 20–30 min after initial formation. However, all aggregates showed very limited movement and eventually located near the cell periphery (Figure 5A). We found that actin depolymerization drugs, such as latrunculin A, made localization less biased to the periphery (Figure 5B), suggesting that actin may play a role in how these aggregates are localized. Yet, it has been shown that actin depolymerization drugs also reduce molecular crowding [41], suggesting that the observed change in localization in the presence of latrunculin A may correspond with changes in molecular crowding rather than a direct effect of the actin cytoskeleton. 

### 4.2. Actin Networks and Aggregate Coalescence

We previously showed that older mothers form multiple aggregates that fail to coalesce compared to their corresponding young daughters [42]. Furthermore, mature propagating [*PSI*^+^] aggregates have been shown to use actin/myosin networks to localize to IPOD structures [43]. Based on these results, we previously proposed that rejuvenated daughters have more robust actin networks that drive coalescence [42]. In this study, we showed that multiple early foci are formed in *act1-120* and *act1-122* strains (Figure 1), and remain separate for several hours, thus providing evidence that properly formed networks are important to aggregate coalescence. However, the lack of direct co-localization between actin and Sup35NM early foci or aggregates further support that any actin effect on potential aggregate coalescence is indirect. Alternatively, it may be that alterations in actin foster the formation of multiple aggregates rather than prevent coalescence. 

### 4.3. Actin and the Transmission of Propagons

Visual Sup35NM-GFP aggregates are hallmarks of prion formation. Cells that contain aggregates of variable size and shape give rise to [*PSI^+^*] cells likely through the transmission of small non-visible infectious prion particles [15,18]. In this study, we found that the multiple aggregates formed in *act1-122* cells (Figure 1D) correlated with decreased prion induction frequency (Figure 6A). Interestingly, *act1-122* has been shown to be associated with prion loss observed with a specific variant of the [*PIN*^+^] prion called μdot. μdot [*PIN*^+^] loss in *act1-122* mutants correlated with the presence of high molecular weight complexes over time [31]. We suspect that prion loss occurred due to increased growth of the aggregate, which could not be passed to daughter cells. While the induction of Sup35NM-GFP has been shown to generate multiple variants [44], we suspect the Sup35 propagons formed in *act1-122* mutants are likely larger than those formed in wildtype cells. Because the size of the propagon dictates the ability to be passed from the mother to the daughter through the bud neck [45], we suspect that a portion of the newly formed Sup35 propagons in *act1-122* cells may grow too large to pass through the bud neck resulting in decreased prion induction frequency.

Heat-induced aggregates and visual prion aggregates have been shown to be retained in the mother cell during cell division [16,46,47,48,49]. This retention has been linked to both Hsp104 and actin networks. We have shown that the disruption of actin cables either through genetic mutation (*act1-101*; Figure 6A) or pharmacological treatment (LatA; Figure 6B) increases prion induction frequency. It is possible that actin cables are important for the retention of prion propagons in the mother cell, thus resulting in lower propagon transmission. However, disruption of actin cables could lead to the transmission of propagons to daughter cells, resulting in the increased prion induction frequency. Taken together, our data shows that actin networks are able to finetune the transmission of newly formed prion aggregates. Further analysis is needed to confirm how actin influences aggregate size and transmission through the bud neck, and whether similar mechanisms are in place for other types of protein aggregates.

## Data Availability

All Python codes and files have been deposited in figshare https://doi.org/10.6084/m9.figshare.19661001.v1; accessed on 26 April 2022.

## Supplementary Materials

The following supporting information can be downloaded at: https://www.mdpi.com/article/10.3390/v14071581/s1, Figure S1: Rhodamine-phalloidin staining of cells expressing Sup35NM-GFP, Figure S2: Sup35NM does not co-localize with Cof1 or Abp140, Figure S3: BY4741 cells have fewer Sup35NM aggregates on average than 74D-694 cells; Figure S4: The movement of early foci is random, Figure S5: Newly formed Sup35NM aggregates do not co-localize with organelles or IPOD, Figure S6: Early foci show sporadic co-localization with Hsp104, but no co-localization with Hsp42; Table S1: Strains used in this study [31,50,51,52,53], Table S2: Plasmids used in this study [14,17,26,27,54,55,56,57,58,59,60]; Video S1: Newly formed early foci in wildtype cells, Video S2: Newly formed early foci in *act1-122* cells.
