# Supplementary material for: Implications of the Actin Cytoskeleton on the Multi-Step Process of [PSI+] Prion Formation
Source: Viruses. 2022 Jul 21;14(7):1581. doi: 10.3390/v14071581 (PMC9321047; doi:10.3390/v14071581)
Supplement: Supplementary file 1 [file viruses-14-01581-s001.zip › viruses-1724381-supplementary materials/viruses-1724381-supplementary-back.pdf]

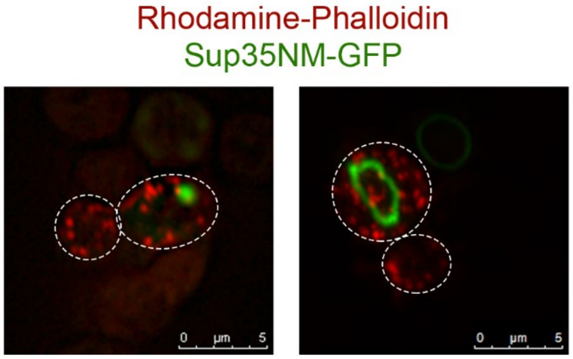

Figure S1. Rhodamine-phalloidin staining of cells expressing Sup35NM-GFP. Wildtype cells expressing Sup35NM-GFP for 24 hours, fixed and stained with rhodamine-phalloidin. Images are representative of multiple cells.

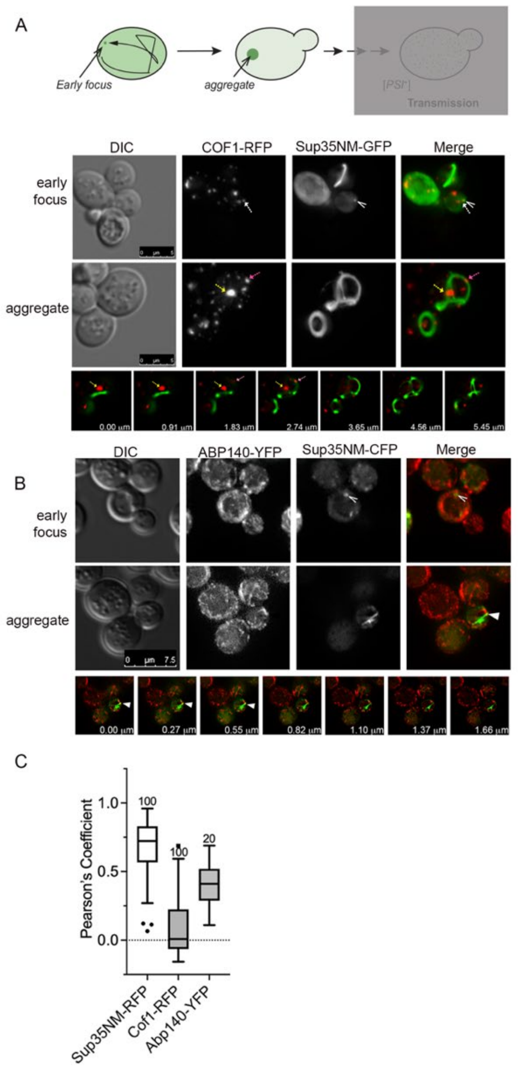

Figure S2. Sup35NM does not co-localize with Cof1 or Abp140. A. Top, model showing multiple steps analyzed in this paper. This figure is focused on the presence of early foci and aggregates. B. 74D-694 wildtype cells with Sup35NM-GFP were grown in the presence of 50uM copper sulfate for 16 hours (top; for the detection of early foci) or 24 hours (bottom; for the detection of aggregates). Cells were imaged for Sup35NM-GFP (green) and Cof1-RFP (red). Open carrot indicates the early focus. The lower panel is a z-stack of the middle panel showing the Cof1-RFP inclusion (yellow dashed arrow) in a different plane than the Sup35NM-GFP aggregate (red dashed arrow). B. Same as A, except Sup35NM-CFP (shown in green) and Abp140-YFP (shown in red) are co-expressed. C. Pearson's correlation coefficient was performed on cells either expressing both Sup35NM-GFP and Sup35NM-RFP (as a positive control) or Sup35NM and the indicated actin fluorescent markers. The open carrot indicates the early focus and the closed arrowhead shows the detectable overlap between ABP140-YFP and Sup35NM-CFP signal.

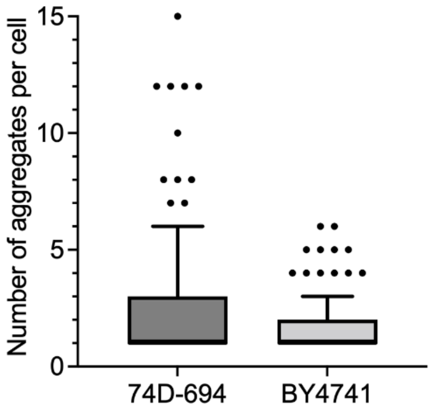

Figure S3. BY4741 cells have fewer Sup35NM aggregates on average than 74D-694 cells. Sup35NM-GFP was expressed in 74D-694 strains for 24 hours or BY4741 strains for 48 hours. The number of aggregates per cell was assessed from over 115 cells. Kolmogorov–Smirnov test shows that the aggregate number distributions are different between strains (p=0.028). Note that for BY4741 strains, all early foci formation was observed after 24 hours of Sup35NM-GFP expression and aggregate formation was observed after 48 hours of Sup35NM-GFP overexpression. Data is shown as a box and whisker plot with median and interquartile ranges. Outliers (shown as dots) were determined using Tukey analysis.

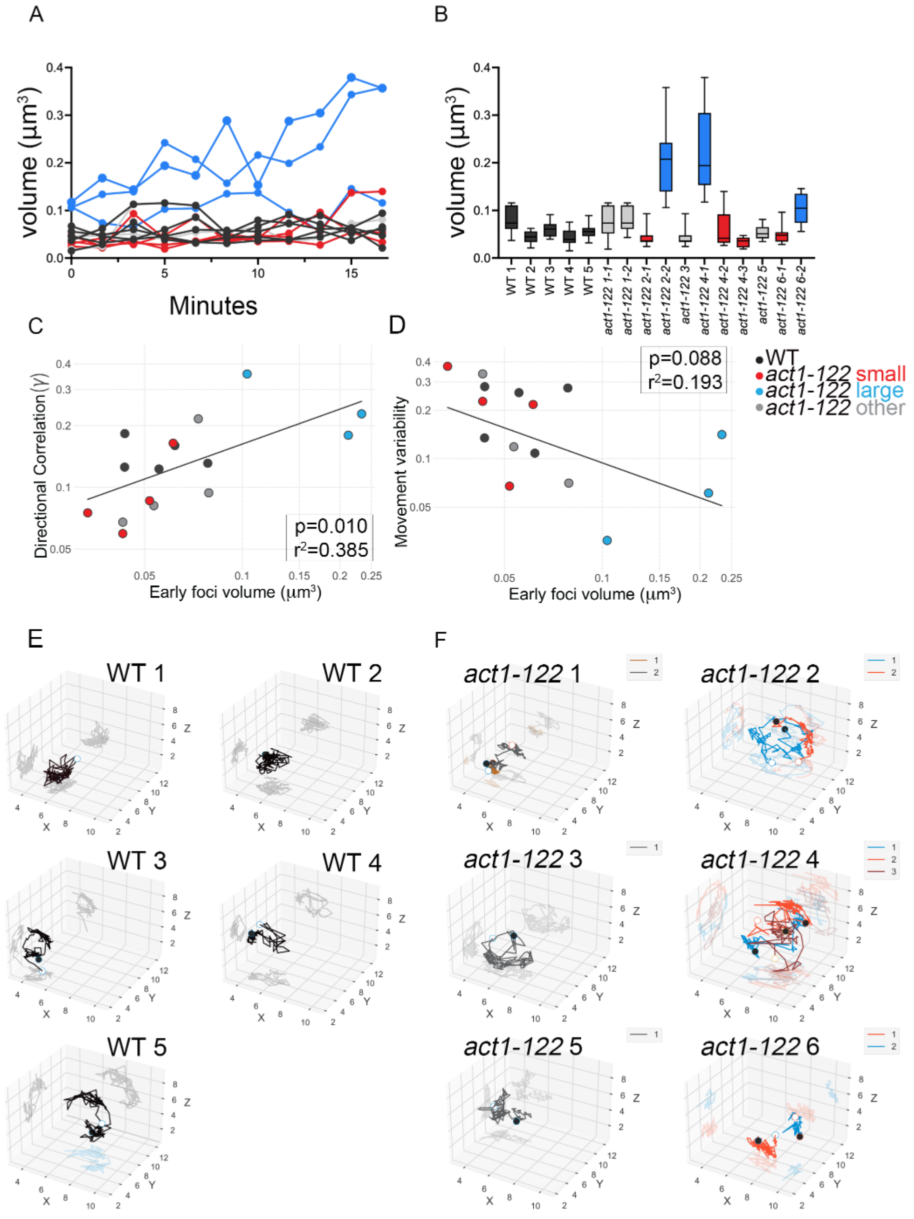

Figure S4. The movement of early foci is random. A. Early foci volume assessed over time in wildtype and *act1-122* cells. B. The average volume of each focus is plotted. C. Directional correlation is plotted against early foci volume. D. Movement variability is plotted against early foci volume. Three-dimensional movement of each focus is shown for five wildtype cells (E) and six *act1-122* cells (F). The color of the trace corresponds to the aggregate number shown in B.

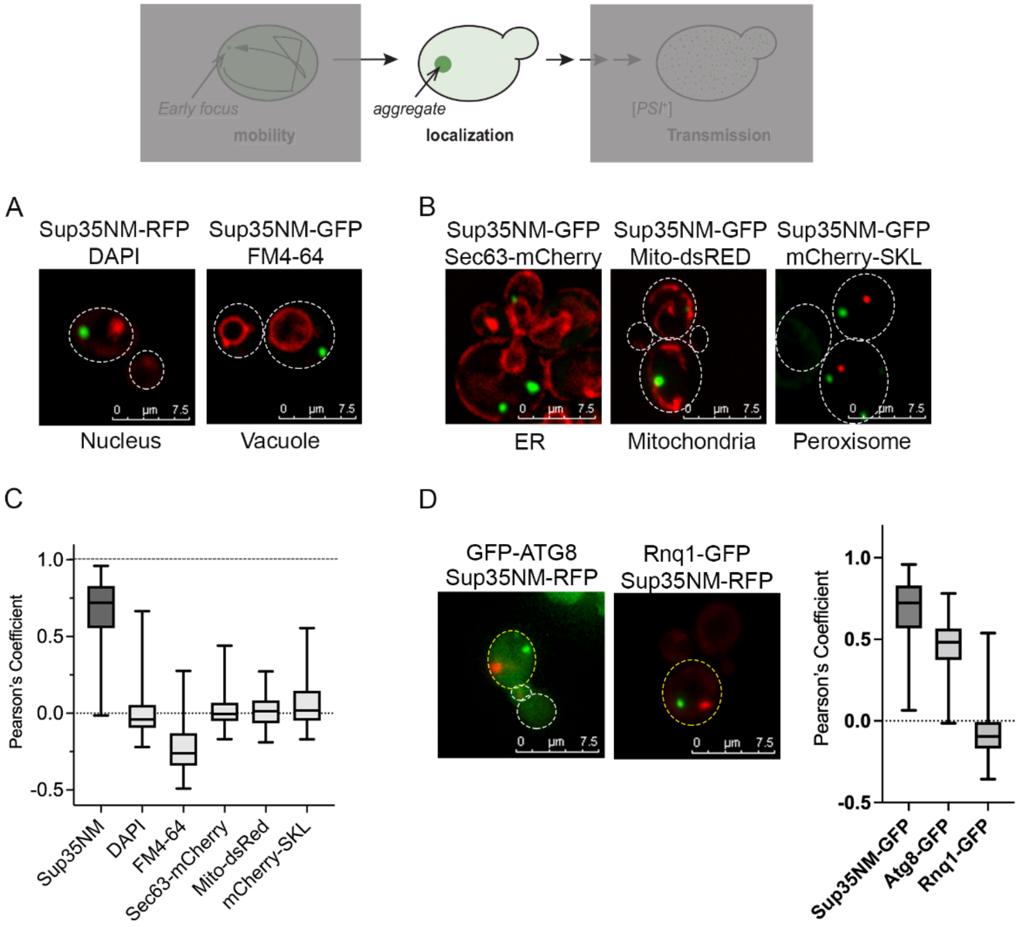

Figure S5. Newly formed Sup35NM aggregates do not co-localize with organelles or IPOD. A. Wildtype cells containing Sup35NM (shown in green) aggregates were treated with DAPI for nuclear staining (left, red) or FM-464 for vacuolar staining (right, red). B. Sup35NM-GFP (green) co-expressed with either Sec63 (left, red) to identify the ER, mito-dsRed (middle, red) to identify the mitochondria, or mCherry-SKL(right, red) to identify peroxisomes, are shown. C. Pearson's correlation coefficient was calculated for all images in A and B. D. Co-localization of Sup35NM-RFP (green) aggregates and GFP-Atg8 (left, red) or Rnq1-GFP (right, red). Pearson's correlation coefficient calculation is shown. Note that co-localization between Sup35NM-GFP and Sup35NM-RFP is used as a control. Data shown in C and D are presented as means ± SD. All values are significantly different from Sup35NM-GFP/Sup35NM-RFP controls (p<0.001) as determined by Welch's t-test, indicating very little co-localization.

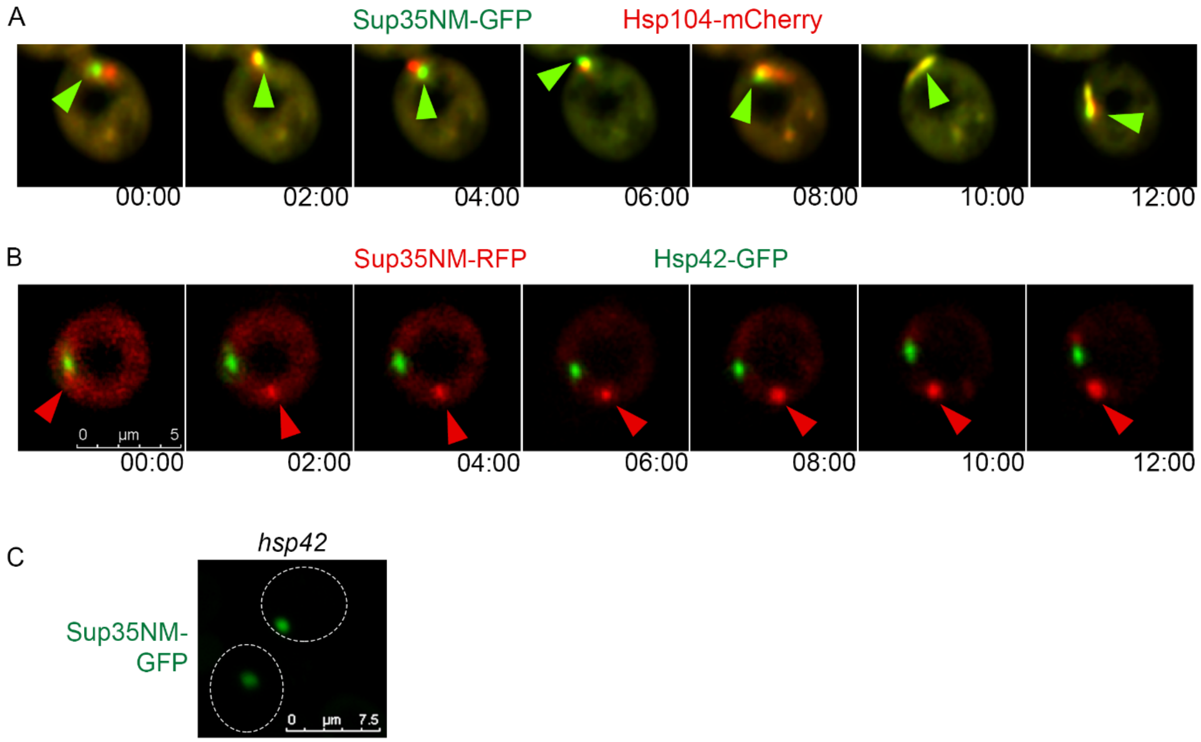

Figure S6. Early foci show sporadic co-localization with Hsp104, but no co-localization with Hsp42. A. 3D time-lapse microscopy was performed with Sup35NM-GFP (green) and Hsp104-mCherry. Green arrows indicate the position of early foci. B. Similar to A, except with Sup35NM-RFP (red) and Hsp42 (green), where red arrows indicate early foci. C. Sup35NM-GFP aggregates are observed in *hsp42Δ* strains.

Supplementary Tables:

Supplemental Table S1. Strains used in this study

**Strain name**	**Genotype**	**Lab number** (Duplicate Strains**)**	**Genetic Background**	**Reference**
Wild type high [*PIN*+]	*MatA his3Δ1; leu2Δ-0; ura3Δ-0; met15Δ-0* high [*PIN*+]	M266 (M102)	BY4741	[1]
Wild type high [*PIN*+]	*MatA ade1-14 ura3-52 leu2-3,112 trp1-289 his3-200* high [*PIN*+]	D233	74D-694	[2]
Abp140-YFP	*MatA ade1-14 leu2-3,112 ura3-52 trp1-289 his3-200 Abp140-YFP(Ura3+)* high [*PIN*+]	M305	74D-694	This study
Wild type low [*PIN*+]	*MatA ade1-14 ura3-52 leu2-3,112 trp1-289 his3-200* low [*PIN*+]	D231	74D-694	[3]
act1-122 high [*PIN*+]	*MatA his3Δ1; leu2Δ-0; ura3Δ-0; act1-122::NATr; MET15; LYS2* high [*PIN*+]	M583 (M600)	BY4741	This study
act1-120 high [*PIN*+]	*MatA his3Δ1; leu2Δ-0; ura3Δ-0; act1-120::NATr; MET15; LYS2* high [*PIN*+]	M310 (M312)	BY4741	[1]
act1-129 [*PIN*+]	*MatA his3Δ1; leu2Δ-0; ura3Δ-0; act1-129::NATr; met15Δ; LYS2* high [*PIN*+]	M258	BY4741	[1]
act1-101 [*PIN*+]	*MatA his3Δ1; leu2Δ-0; ura3Δ-0; act1-101::NATr; met15Δ; LYS2* high [*PIN*+]	M256	BY4741	[1]
Hsp104-GFP	*MatA ura3 leu2 his3 met15 Hsp104-GFP (YLL026w);* library [*PIN*+]	D228	BY4741	[4]
Hsp42-GFP	*MatA ura3 leu2 his3 met15 Hsp42-GFP (YDR171w);* library [*PIN*+]	D229	BY4741	[4]
Ssa1-GFP	*MatA ade1-14 ura3-52 leu2-3,112 trp1-289 his3-200 Ssa1-GFP::KANMX6* high [*PIN*+]	M262	74D-694	[5]
Sis1-GFP	*MatA ade1-14 ura3-52 leu2-3,112 trp1-289 his3-200 Sis1-GFP::KANMX6* high [*PIN*+]	M261	74D-694	[5]
hsp42D	*MatA ade1-14 leu2-3,112 ura3-52 trp1-289 his3-200 hsp42::HIS3* high [*PIN*+]	M383	74D-694	This study
act1-122 μdot [*PIN*+]	*MatA his3Δ1; leu2Δ-0; ura3Δ-0; act1-122::NATr; MET15; LYS2* μdot [*PIN*+]	M254	BY4741	[1]

Table S2. Plasmids used in this study

Plasmid name	Lab number	Yeast Markers	Reference
pCUP-RNQ1-GFP	*p3036*	*LEU2, CEN*	[6]<br>Fig. S7
pCUP-SUP35NM-GFP	*p3031*	*HIS3, CEN*	[7]<br>Fig. 1-4, 6<br>Fig. S4-6
pCUP-SUP35NM-GFP	*p3032*	*LEU2, CEN*	[7]<br>Fig. 5<br>Fig. S1-3, 7
pRS416-pCOF1-COF1RFP	*p3069*	*URA3, CEN*	[8]<br>Fig. S2
pABP1-ABP140-3XYFP	*p3089*	*URA3, Integrating*	[9]<br>Fig. S2
pCUP-Sup35NM:CFP	*p3053*	*HIS3, CEN*	[10]<br>Fig. S2
pCUP-Sup35NM-RFP	*p3121*	*URA3, CEN*	[11]<br>Fig. 4<br>Fig. S7-8
pRS416-pSec63-Sec63-mCherry-Tcyc1	*p3165*	*URA3, CEN*	[12]<br>Fig. S7
pYX142 mito-dsRed	*p3162*	*LEU2, CEN*	[13]<br>Fig. S7
pRS416-pGPD-mCherry-SKL-Tcyc1	*p3167*	*URA3, CEN*	[12]<br>Fig. S7
pCUP-GFP-ATG8	*p3068*	*URA3, CEN*	[14]<br>Fig. S7
pAG415-GPD-Hsp104-mCherry	*p3172*	*LEU2, CEN*	[15]<br>Fig. 4<br>Fig. S8
pLEU2ura3-14	*p3107*	*LEU2, CEN*	[16]<br>Fig. 6

pCUP is a copper-inducible promoter. GPD is a constitutive promoter.

Reference

1. Dorweiler, J.E.; Oddo, M.J.; Lyke, D.R.; Reilly, J.A.; Wisniewski, B.T.; Davis, E.E.; Kuborn, A.M.; Merrill, S.J.; Manogaran, A.L. The actin cytoskeletal network plays a role in yeast prion transmission and contributes to prion stability. *Mol. Microbiol.* **2020**, *114*, 480–494. https://doi.org/10.1111/mmi.14528.
2. Derkatch, I.L.; Bradley, M.E.; Zhou, P.; Chernoff, Y.O.; Liebman, S.W. Genetic and environmental factors affecting the de novo appearance of the [PSI+] prion in Saccharomyces cerevisiae. *Genetics* **1997**, *147*, 507–519.
3. Bradley, M.E.; Edskes, H.K.; Hong, J.Y.; Wickner, R.B.; Liebman, S.W. Interactions among prions and prion "strains" in yeast. *Proc. Natl. Acad. Sci. USA* **2002**, *99* (Suppl. 4), 16392–16399. https://doi.org/10.1073/pnas.152330699.
4. Huh, W.K.; Falvo, J.V.; Gerke, L.C.; Carroll, A.S.; Howson, R.W.; Weissman, J.S.; O'Shea, E.K. Global analysis of protein localization in budding yeast. *Nature* **2003**, *425*, 686–691. https://doi.org/10.1038/nature02026.
5. Klaips, C.L.; Hochstrasser, M.L.; Langlois, C.R.; Serio, T.R. Spatial quality control bypasses cell-based limitations on proteostasis to promote prion curing. *eLife* **2014**, *3*, e04288. https://doi.org/10.7554/eLife.04288.
6. Sondheimer, N.; Lindquist, S. Rnq1: An epigenetic modifier of protein function in yeast. *Mol. Cell* **2000**, *5*, 163–172.

7. Zhou, P.; Derkatch, I.L.; Liebman, S.W. The relationship between visible intracellular aggregates that appear after overexpression of Sup35 and the yeast prion-like elements [PSI(+)] and [PIN(+)]. *Mol. Microbiol.* **2001**, *39*, 37–46, doi:mmi2224 [pii].
8. Lin, M.C.; Galletta, B.J.; Sept, D.; Cooper, J.A. Overlapping and distinct functions for cofilin, coronin and Aip1 in actin dynamics in vivo. *J. Cell Sci.* **2010**, *123*, 1329–1342. https://doi.org/10.1242/jcs.065698.
9. Buttery, S.M.; Yoshida, S.; Pellman, D. Yeast formins Bni1 and Bnr1 utilize different modes of cortical interaction during the assembly of actin cables. *Mol. Biol. Cell* **2007**, *18*, 1826–1838. https://doi.org/10.1091/mbc.E06-09-0820.
10. Du, Z.; Valtierra, S.; Li, L. An insight into the complex prion-prion interaction network in the budding yeast Saccharomyces cerevisiae. *Prion* **2014**, *8*, 387–392. https://doi.org/10.4161/19336896.2014.992274.
11. Arslan, F.; Hong, J.Y.; Kanneganti, V.; Park, S.K.; Liebman, S.W. Heterologous aggregates promote de novo prion appearance via more than one mechanism. *PLoS Genet.* **2015**, *11*, e1004814. https://doi.org/10.1371/journal.pgen.1004814.
12. Wang, C.W.; Miao, Y.H.; Chang, Y.S. Control of lipid droplet size in budding yeast requires the collaboration between Fld1 and Ldb16. *J. Cell Sci.* **2014**, *127*, 1214–1228. https://doi.org/10.1242/jcs.137737.
13. Naylor, K.; Ingerman, E.; Okreglak, V.; Marino, M.; Hinshaw, J.E.; Nunnari, J. Mdv1 interacts with assembled dnm1 to promote mitochondrial division. *J. Biol. Chem.* **2006**, *281*, 2177–2183. https://doi.org/10.1074/jbc.M507943200.
14. Wang, K.; Yang, Z.; Liu, X.; Mao, K.; Nair, U.; Klionsky, D.J. Phosphatidylinositol 4-kinases are required for autophagic membrane trafficking. *J. Biol. Chem.* **2012**, *287*, 37964–37972. https://doi.org/10.1074/jbc.M112.371591.
15. Malinovska, L.; Kroschwald, S.; Munder, M.C.; Richter, D.; Alberti, S. Molecular chaperones and stress-inducible protein-sorting factors coordinate the spatiotemporal distribution of protein aggregates. *Mol. Biol. Cell* **2012**, *23*, 3041–3056. https://doi.org/10.1091/mbc.E12-03-0194.
16. Manogaran, A.L.; Kirkland, K.T.; Liebman, S.W. An engineered nonsense URA3 allele provides a versatile system to detect the presence, absence and appearance of the [PSI+] prion in Saccharomyces cerevisiae. *Yeast* **2006**, *23*, 141–147. https://doi.org/10.1002/yea.1341.
